# Biliary ascariasis without cholangitis: an unusual presentation in an endemic region

**DOI:** 10.1093/omcr/omaf268

**Published:** 2025-12-26

**Authors:** Nabil, Muhammad Ashar Zohaib, Muhammad Zubair, Urvah Shafique, Javed Iqbal, Asraf Hussain

**Affiliations:** Department of Medicine, Allama Iqbal Medical College, Jinnah Hospital, Lahore, Punjab 54700, Pakistan; Department of Medicine, Allama Iqbal Medical College, Lahore, Punjab 54700, Pakistan; Department of Medicine, Allama Iqbal Medical College, Lahore, Punjab 54700, Pakistan; Department of Medicine, Allama Iqbal Medical College, Lahore, Punjab 54700, Pakistan; Nursing Department Hamad Medical Corporation Doha, Department of Nursing, P.O Box 3050, Doha, Qatar; Chitwan Medical College, Chitwan Bharatpur Nepal, Bharatpur, P.O Box 44200, Nepal

**Keywords:** Ascaris lumbricoides, biliary ascariasis, parasitic infestation, MRCP, hepatobiliary disorders, ERCP

## Abstract

Biliary ascariasis is a clinical condition, in which the roundworm Ascaris lumbricoides migrates from the small intestine into the biliary tract. We report a case of a 21-year-old female patient from Skardu, Pakistan. The patient shows an unusual presentation that makes the diagnosis quite tricky. The symptoms include dull, persistent right hypochondrial pain, nausea, and vomiting without any signs of jaundice or fever. Moreover, the blood tests revealed only a mild increase in eosinophil count, just above the borderline, and completely normal liver function, a distinguishing feature of parasitic infections. Ultrasound showed a dilated bile duct containing a roundworm, indicating a parasite infection. Initially, the patient was given deworming medication. Furthermore, Magnetic resonance cholangiopancreatography (MRCP) findings were consistent with biliary ascariasis characterized by the presence of Ascaris within the hepatic biliary channels causing its significant dilatation. This case report highlights that lab reports can sometimes be misleading when making a diagnosis of biliary ascariasis. Additionally, there is a need for improvement in the healthcare system and sanitation.

## Introduction

Ascariasis is a helminthic infection caused by the roundworm *Ascaris lumbricoides*, one of the most transmitted parasitic infections all over the world. The parasite is transmitted via the fecal or oral route, mainly in endemic regions with poor sanitation infrastructure. After ingestion, the larvae hatch and mature into adult worms in the small intestine. Moreover, it remains clinically silent until it migrates into the biliary tract. Biliary tract disorders are predominantly caused by cholelithiasis (gallstones). However, in regions endemic to parasitic infestations or areas with poor hygiene and sanitation, hepatobiliary disorders may be due to an underlying worm infestation [1].

**Figure 1:**
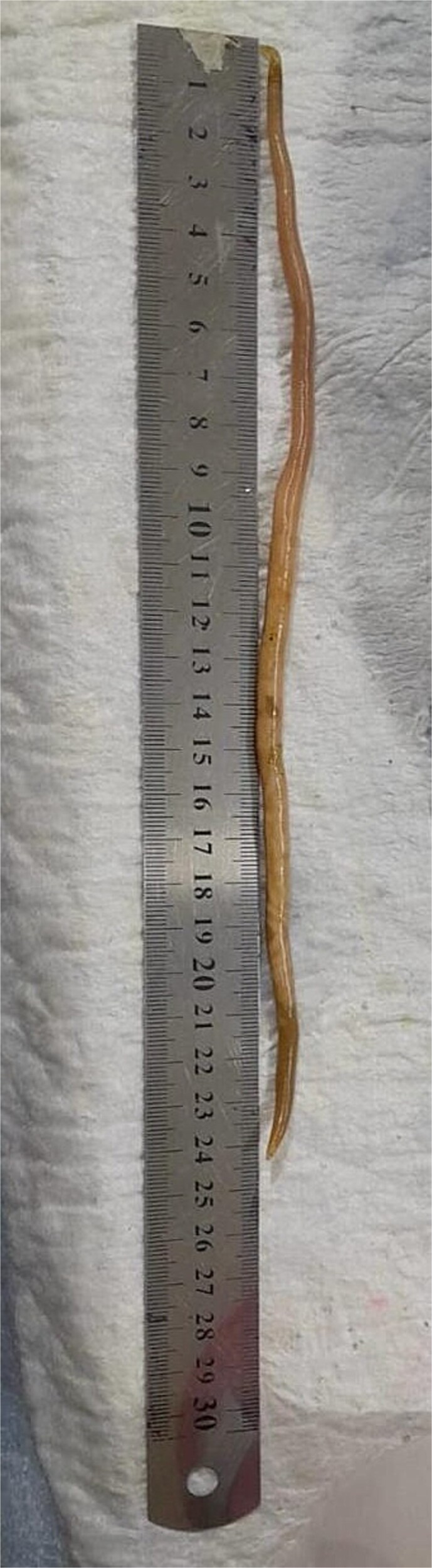
Measurement of the parasitic worm post extraction.

**Figure 2:**
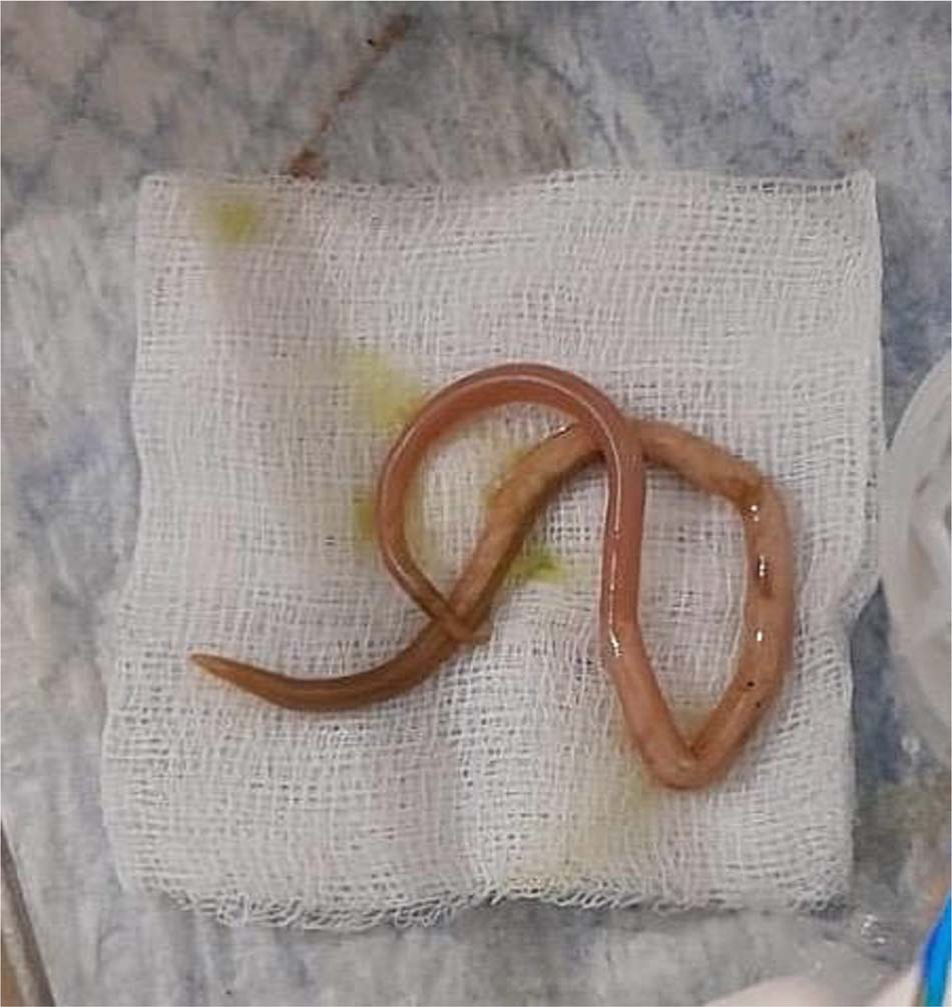
Extracted parasitic worm.

**Figure 3:**
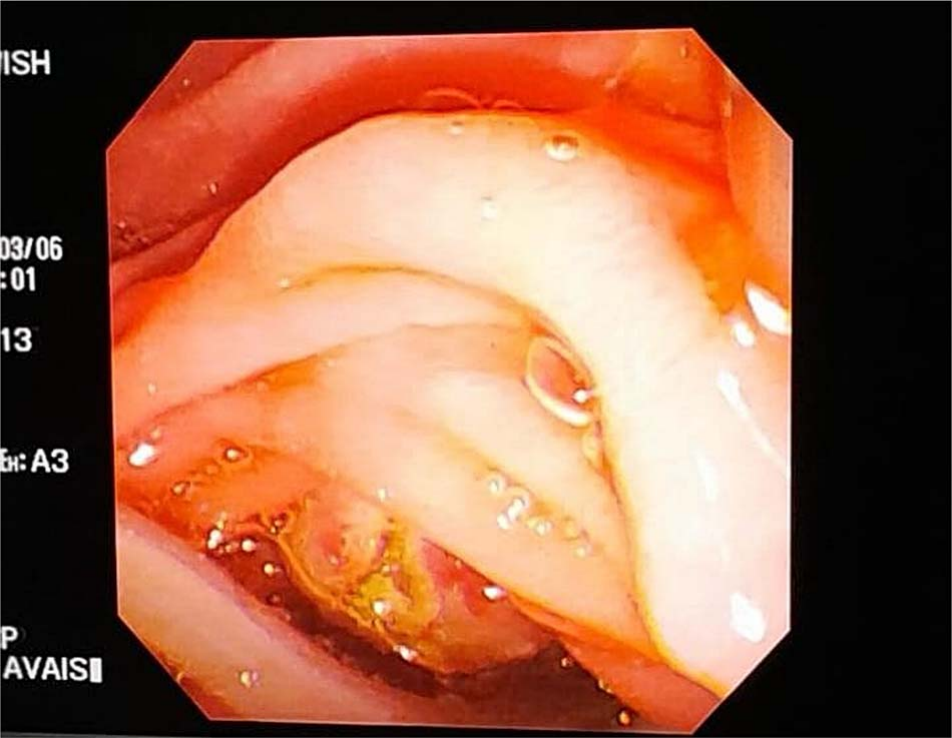
Endoscopic view of the gastrointestinal tract showing a parasitic worm.

This intestinal nematode mainly inhabits the duodenum or jejunum but can migrate through the ampulla of Vater into the biliary tree. [2, 3] Its presence in the biliary system can cause a variety of complications such as cholangitis, liver abscesses, choledocholithiasis, and pancreatitis. [4] Biliary ascariasis is more common in endemic regions such as Ethiopia and South Asia, including rural areas of Pakistan. [5] Furthermore, in endemic areas, 30% of adults are affected. It has been more frequently reported in females, mainly due to anatomical and physiological factors. [6] We report a case of an adult female with the less frequent presentation of biliary ascariasis (Figs 1–3).

## Case presentation

A young female patient from a high-endemic region in Pakistan, having no chronic medical illness, presented to Jinnah Hospital Lahore with a history of right hypochondriac pain. The pain was dull in nature, non-radiating, and worsening with movements and changes in position. Additionally, it was unresponsive to analgesics or antacids. It started gradually without a specific pattern, and many times reached 10/10 in severity. She also experienced anorexia for one month, nausea, and vomiting for a day’s duration. However, the patient denied any history of jaundice, diarrhea, constipation, hematemesis, and melena. Her vitals were within normal limits.

On examination, the patient was conscious and oriented. However, her abdomen was soft and lax, with severe epigastric tenderness without any palpable mass or hepatosplenomegaly. She had a previous history of intestinal ascariasis two years back, for which she had taken a single dose of albendazole.

Laboratory tests were performed on 24^th^ February, shortly after the patient first visited the hospital, and were repeated on 27^th^ February. Notably, the eosinophils, liver function tests and leukocytes remained within the normal limits. However, marked increase in monocytes was reported. Abdomen Ultrasound was done, which showed a dilated common bile duct (CBD), measuring 9 mm. There was a linear tubular echogenic structure noted in the common bile duct. However, other major visualized abdominal organs were unremarkable. Magnetic resonance cholangiopancreatography (MRCP) was characterized by the presence of ascaris within the intra- and extrahepatic biliary channels, causing significant dilatation. However, the laboratory tests showed that eosinophil count remained within the normal limits, this was misleading because in parasitic infections, eosinophilia is typically observed. This unusual presentation made the diagnosis more challenging and contributed to uncertainty.

**Table 1:** 

Test	Result 27^th^ February 04:58 PM	Result 24^th^ February 07:09 PM	Normal Range
WBC	14.7 ↑	13.4 ↑	4–11 × 10^9^/L
HGB (Hemoglobin)	12.1	12.8	12–15 g/dL
HCT (Hematocrit)	37.5	34.7 ↓	36–46%
MCH	28.8	33.2 ↑	27–31 pg
MCHC	32.2	36.8 ↑	31.5–34.5 g/dL
MCV	89.7	90.2	76–96 fL
PLT	268	270	150–450 × 10^9^/L
%Neutrophils	36.1 ↓	38.7 ↓	40–80%
%Lymphocytes	23.4	23.9	20–40%
%Monocytes	35.3 ↑	30.4 ↑	2–8%
%Eosinophil	5	6	1–6%
AST	35	25	10–40 U/L
ALT	37	21	10–40 U/L
Total Bilirubin	0.3	0.5	0.1–1.20 mg/dL

Initially, choledocholithiasis was considered because of right hypochondrial pain and common bile duct dilatation. However, this was excluded from diagnosis because lab reports showed normal bilirubin and no gallstones were present. The patient had no fever or jaundice, so acute cholangitis was ruled out. Moreover, acute pancreatitis was also considered due to epigastric pain, but lack of pain radiation to the back, normal liver function tests and absence of pancreatic enzymes elevation decreased the probability. Similarly, abdominal pain was not responsive to antacids or analgesics, it mitigated the risk of peptic ulcer disease. No guarding was observed in the abdomen, and it was soft and lax which suggested that peritonitis is not the diagnosis. The presence of CBD dilatation with a linear tubular echogenic structure supported the diagnosis of biliary ascariasis. MRCP results further confirmed the diagnosis.

## Discussion

This 21-year-old female, from the rural area, was diagnosed with biliary ascariasis. The most unusual aspect of this case was the normal laboratory profile: liver function tests were near normal and there was no eosinophilia, despite confirmed biliary ascariasis. Although she experienced mild right hypochondrial pain, nausea, and occasional vomiting, these symptoms are typical and not the primary unusual feature. Most reports describe fever, jaundice, and raised bilirubin due to biliary obstruction [6], and studies from endemic regions like South Asia often show cholangitis as the classical presentation [7, 8], emphasizing that it was the normal labs that made this presentation atypical. Specifically, the lack of eosinophilia and a normal set of liver function tests in the face of confirmed infection renders this case atypical. However, a history of previous intestinal ascariasis was an important clinical clue. This is a demonstration of the importance of taking good history in making a diagnosis, especially in situations where atypical presentations are made.

Biliary ascariasis is rare globally but is more prevalent in developing nations with inadequate sanitation and hygiene [6, 7]. In rural Pakistan, for instance, Ascaris infection afflicts as many as 30% of adults, although all of them do not develop pathology in the liver or bile ducts [6]. Our patient had no underlying chronic health status, but she resided in a community with limited clean water and open defecation prevalent. The gender, though, has some studies indicating that females are a bit more at risk due to hormonal fluctuations, particularly progesterone, causing the sphincter of Oddi to relax and allowing easier passage of worms into the bile ducts [5]. Her environmental exposure along with this might explain her presentation. Her history is consistent with what has been reported in local studies, in which parasitic infections are more prevalent among the low-income and poor living conditions [9]. All of these indicate how significant it is to consider the residential area, living condition and exposure risk of the patient when coming up with a diagnosis.

Ultrasonography was the initial investigation in our case and was found to be a dilated common bile duct containing a tubular echogenic structure, which is a characteristic appearance in biliary ascariasis [10]. This so-called ‘strip sign’ or ‘spaghetti sign’ is well described in imaging literature as highly suggestive of live worms within the biliary system [10, 11]. In contrast to the radiological mimics such as biliary sludge or choledocholithiasis, the mobile linear pattern of the structure in our patient’s scan was decisive in establishing the parasitic origin of the obstruction. Ultrasonography, together with a history of prior intestinal ascariasis, was sufficient to establish the diagnosis, showing the characteristic dilated bile duct with a tubular echogenic structure. MRCP was subsequently performed only to confirm the finding. This highlights the value of ultrasound in resource-limited settings, where MRCP may not be readily available. While ERCP is often used for both diagnosis and treatment in complicated cases, in our patient it was performed only therapeutically to remove the worm after medical therapy, as the diagnosis had already been established by ultrasound and confirmed by MRCP. The patient was started on antihelminthic medications, which improved symptoms, and later ERCP was carried out to retrieve the worm. Indian and Iranian studies have also reported similar ultrasound findings, confirming that in skilled hands, sonography is still a sound tool for diagnosing biliary ascariasis [11].

Treatment began with oral albendazole 400 mg, which settled her symptoms. Five days later, ERCP was done, and a 24 cm worm was retrieved. This finding is testimony to the success of anthelmintic therapy in uncomplicated or early biliary ascariasis. ERCP is recommended in standard protocols for complicated or severely symptomatic cases, like obstruction and cholangitis [8]. Although there were no signs of infection, ERCP was chosen because of the dilated duct and visible worm, making it the safest way to secure definitive clearance. No post-treatment complication or recurrence was noted during follow-up, concordant with other reports of successful treatment following single dose albendazole therapy followed by ERCP. [12]

The case also demonstrates more widespread concerns of rural or under-supported district patients. In under-supported and rural districts, diagnosis tools like ultrasound, MRCP, or ERCP are usually postponed because of closeness and availability. The patient had to travel to big cities to guarantee diagnosis and treatment show of how clinical results are affected by healthcare inequalities. In most such comparable cases, delayed access may lead to complications that require invasive procedures or hospitalization. Success in diagnosis in the absence of using advanced imaging or emergency treatment is a respite but also an indication of the need for augmenting healthcare infrastructure in endemic regions. Such elementary steps like training sonographers, increasing equipment availability, and implementing routine deworming programs can be highly effective.

This case is significant because it illustrated a different presentation for biliary ascariasis, without the typical lab findings most physicians are used to. When we compared our patient’s lab findings with reported cases, we were able to see how variable the disease could be. That made us better understand the condition. It also demonstrated the value of not forgetting parasitic etiologies when the diagnosis remains uncertain. Reporting such cases contributes to the scarce information from areas such as ours and can serve as a guide to physicians who might encounter similar patients in the future. Early suspicion and simple imaging can be the key.

## Conclusion

The patient had a rare presentation of biliary ascariasis with no clinical evidence of cholangitis. Absence of typical signs such as fever and jaundice made this case a rare one. The diagnosis was established based on ultrasound and MRCP findings providing a non-invasive and precise way to identify worm infestations of the biliary system. It effectively reduces the dependence on ERCP and its associated risks, reserving ERCP for therapeutic interventions. This interesting presentation means that parasitic biliary disease still must be considered in differential diagnosis, particularly in patients from endemic areas, even when classical presentation is not evident. It also means that the medical centers in endemic areas should be well equipped to do ultrasound and MRCP. This will be helpful in early diagnosis and avoidance of some of the complications such as unnecessary invasive procedures. Clinicians must be aware of such unusual presentations too and consider biliary ascariasis in the differential diagnosis, to maximize patient care.

## References

[1] Rokaitė R, Dženkaitis M, Nedzinskaitė M. et al. Biliary ascariasis in a Pediatric patient in Lithuania: case report and literature review. Med Kaunas Lith 2024;60:916. 10.3390/medicina60060916 PMC11205314 38929533

[2] Akbar I, Javed Z, Zaib Z. Gall bladder ascariasis: a rare entity. J Ayub Med Coll Abbottabad JAMC 2023;35:500–2. 10.55519/JAMC-03-10212 38404103

[3] Kolleri JJ, Thabet AMJ, Mohammedain S. et al. A case report on biliary ascariasis. Cureus 2023;15:e33323. 10.7759/cureus.33323 36741635 PMC9894724

[4] Reddy S, Boloor A, Thomas NK. An interesting case of cholangitis. Cureus 2024;16:e60537. 10.7759/cureus.60537 38887340 PMC11181122

[5] Asfaw YA, Asrat GT, Uddo TB. et al. Successful Management of Biliary Ascariasis in a high-endemic zone and low-resource setting in Ethiopia. Case Rep Infect Dis 2022;2022:1–4. 10.1155/2022/8201398 PMC9733989 36504673

[6] Rujeerapaiboon N, Kaewdech A. Massive biliary ascariasis: an unusual cause of acute cholangitis. BMJ Case Rep 2021;14:e239784. 10.1136/bcr-2020-239784 PMC7993313 33762275

[7] Inyang B, Koshy FS, George K. et al. An overview of ascariasis involvement in gallbladder disease: a systematic review of case reports. Cureus 2022;14:e32545. 10.7759/cureus.32545 36654632 PMC9840414

[8] Patra PS, Das A, Ahmed SKM. et al. Treatment response and long-term outcomes in biliary ascariasis: a prospective study. Arab J Gastroenterol 2021;22:164–9. 10.1016/j.ajg.2020.11.002 33752976

[9] Rahimi M, Mohamad IS, Yahya MM. et al. Biliary ascariasis: extraction. Endoscopy 2024;56:E195–6. 10.1055/a-2258-8436 38388953 PMC10883872

[10] Che Husin N, Mohamad IS, Ho KY. et al. Biliary ascariasis - a vicious cycle. Malays Fam Physician 2021;16:83–5. 10.51866/cr1078 34386170 PMC8346760

[11] Demessie AG, Ayalew S, Negussie MA. et al. Gallbladder perforation due to biliary ascariasis: a case report. Int J Surg Case Rep 2024;125:110536. 10.1016/j.ijscr.2024.110536 39476717 PMC11550615

[12] Silva C, Gonçalves IC, Neves S. et al. Diagnosis of asymptomatic biliary ascariasis by abdominal ultrasound in a non-endemic area. Cureus 2023;15:e33599. 10.7759/cureus.33599 36788831 PMC9910813

